# Etp1 confers arsenite resistance by affecting *ACR3* expression

**DOI:** 10.1093/femsyr/foac018

**Published:** 2022-03-22

**Authors:** Antonia M Romero, Ewa Maciaszczyk-Dziubinska, Mandana Mombeinipour, Emma Lorentzon, Emelie Aspholm, Robert Wysocki, Markus J Tamás

**Affiliations:** Department of Chemistry and Molecular Biology, University of Gothenburg, S-405 30 Göteborg, Sweden; Faculty of Biological Sciences, University of Wroclaw, 50-328 Wroclaw, Poland; Department of Chemistry and Molecular Biology, University of Gothenburg, S-405 30 Göteborg, Sweden; Department of Chemistry and Molecular Biology, University of Gothenburg, S-405 30 Göteborg, Sweden; Department of Chemistry and Molecular Biology, University of Gothenburg, S-405 30 Göteborg, Sweden; Faculty of Biological Sciences, University of Wroclaw, 50-328 Wroclaw, Poland; Department of Chemistry and Molecular Biology, University of Gothenburg, S-405 30 Göteborg, Sweden

**Keywords:** arsenic, metalloid, Yap8, Etp1, Acr3

## Abstract

In a high-throughput yeast two-hybrid screen of predicted coiled-coil motif interactions in the *Saccharomyces cerevisiae* proteome, the protein Etp1 was found to interact with the yeast AP-1-like transcription factors Yap8, Yap1 and Yap6. Yap8 plays a crucial role during arsenic stress since it regulates expression of the resistance genes *ACR2* and *ACR3*. The function of Etp1 is not well understood but the protein has been implicated in transcription and protein turnover during ethanol stress, and the *etp1∆* mutant is sensitive to ethanol. In this current study, we investigated whether Etp1 is implicated in Yap8-dependent functions. We show that Etp1 is required for optimal growth in the presence of trivalent arsenite and for optimal expression of the arsenite export protein encoded by *ACR3*. Since Yap8 is the only known transcription factor that regulates *ACR3* expression, we investigated whether Etp1 regulates Yap8. Yap8 ubiquitination, stability, nuclear localization and *ACR3* promoter association were unaffected in *etp1∆* cells, indicating that Etp1 affects *ACR3* expression independently of Yap8. Thus, Etp1 impacts gene expression under arsenic and other stress conditions but the mechanistic details remain to be elucidated.

## Teaser

Etp1 confers arsenite resistance in *Saccharomyces cerevisiae* by affecting *ACR3* expression in a Yap8-independent fashion.

## Introduction

Cells continuously monitor and respond to changes in their internal and external environment. For instance, the yeast *Saccharomyces cerevisiae* rapidly regulates gene expression, protein synthesis and metabolism in response to stress conditions, including exposure to toxic metals, oxidative stress, high temperature and fluctuations in osmolarity. These responses are important for survival in harsh conditions as well as for growth and proliferation in suboptimal environments (Hohmann and Mager 1997).

The metalloid arsenic is highly toxic and abundant in the environment. In humans, chronic arsenic exposure causes cancers of the skin, lung, bladder, kidney and liver, and is associated with various neurodegenerative disorders, cardiovascular disease, hypertension and diabetes. The molecular mechanisms underlying the toxic and carcinogenic effects of arsenic are not well understood but may involve binding to and inactivation of specific enzymes, induction of oxidative and proteotoxic stress, inhibition of DNA repair systems, deregulation of cell proliferation, changes to the epigenome and interference with signal transduction pathways (Shen *et al*. 2013; Tamás *et al*. 2014; Zhou and Xi 2018). Microorganisms are also exposed to arsenic and past studies in *S. cerevisiae* uncovered several toxicity and resistance mechanisms, including transport proteins that mediate arsenic influx or catalyze its export, intracellular and extracellular chelation mechanisms that prevent arsenic influx or protect the intracellular environment from toxic arsenic interactions, and protein quality control systems that safeguard the integrity of the proteome during exposure. In several cases, similar mechanisms have been shown to operate in higher eukaryotes (Rosen and Tamás 2010; Wysocki and Tamás 2010, 2011; Maciaszczyk-Dziubinska, Wawrzycka and Wysocki 2012).

Arsenic can enter cells in form of pentavalent arsenate [As(V)] through phosphate transporters (Bun-ya *et al*. 1996; Yompakdee *et al*. 1996; Shen *et al*. 2012), or in form of trivalent arsenite [As(III)] through aquaglyceroporins (Wysocki *et al*. 2001) and hexose transporters (Liu, Boles and Rosen 2004). The arsenate reductase Acr2 (also called Arr2) converts intracellular As(V) to As(III) (Mukhopadhyay and Rosen 1998; Mukhopadhyay, Shi and Rosen 2000) and intracellular As(III) can be exported out of cells by the plasma membrane protein Acr3 (also called Arr3) (Wysocki, Bobrowicz and Ulaszewski 1997) or into vacuoles via the ABC transporter Ycf1 (Ghosh, Shen and Rosen 1999). *Saccharomyces cerevisiae* responds to As(III) by increasing the production of the tri-peptide glutathione for intracellular and extracellular chelation and detoxification (Thorsen *et al*. 2007, 2012; Talemi *et al*. 2014). Additionally, intracellular As(III) can be methylated by the methyltransferase Mtq2 and the presence of methylarsenite appears to elicit distinct protective responses (Lee and Levin 2018, 2019, 2022). The systems above are regulated at transcriptional and posttranslational levels. For example, transcriptional activation of detoxification genes involves Yap8 (also called Acr1 and Arr1) that controls expression of *ACR2* and *ACR3* (Menezes *et al*. 2004; Wysocki *et al*. 2004; Kumar *et al*. 2016), Yap1 that regulates *YCF1* expression (Wysocki *et al*. 2004) and Met4 that together with Yap1 regulates expression of glutathione biosynthesis-related genes (Wysocki *et al*. 2004; Thorsen *et al*. 2007). Similarly, posttranslational regulation of arsenic transporters affects intracellular arsenic concentration and resistance (Thorsen *et al*. 2006; Beese, Negishi and Levin 2009; Ahmadpour *et al*. 2016; Lee and Levin 2018; Jochem *et al*. 2019; Wawrzycka *et al*. 2019; Lee and Levin 2022). Widespread protein misfolding and aggregation contributes to the toxicity of As(III) (Jacobson *et al*. 2012; Andersson *et al*. 2021) and As(III)-exposed cells decrease global protein synthesis and increase the protein degradation capacity to enhance resistance (Jacobson *et al*. 2012; Guerra-Moreno *et al*. 2015; Andersson *et al*. 2021). The broad range of responses launched by yeast cells to arsenic stress is probably a reflection of the pleiotropic nature of the toxicity of this metalloid.

Yap8 is a member of the yeast AP-1-like (yAP) transcription factor family (Rodrigues-Pousada *et al*. 2019) and it plays a crucial role during arsenic stress by regulating expression of the arsenic resistance genes *ACR2* and *ACR3* (Menezes *et al*. 2004, 2008; Wysocki *et al*. 2004; Kumar *et al*. 2016). These genes are transcribed in opposite directions from a shared promoter that contains a Yap8-binding element (Wysocki *et al*. 2004; Ilina *et al*. 2008; Maciaszczyk-Dziubinska *et al*. 2020). Yap8 is bound to the *ACR2-ACR3* promoter both in the absence and presence of As(III). Direct binding of As(III) to specific cysteine residues within Yap8 induces a conformational change that converts inactive Yap8 into an active transcriptional regulator (Wysocki *et al*. 2004; Di and Tamás 2007; Kumar *et al*. 2016). Additionally, Yap8 is phosphorylated by the Hog1 kinase during As(III) stress and this phosphorylation contributes to efficient induction of *ACR2* and *ACR3* expression by an unknown mechanism (Guerra-Moreno *et al*. 2019). Full induction of *ACR2* and *ACR3* expression also involves coactivator complexes and chromatin remodeling factors (Menezes *et al*. 2017; West *et al*. 2019). Although Yap8 and *ACR3* regulation have been intensely studied, several questions remain unanswered regarding how Yap8 couples arsenic sensing to transcriptional activation of *ACR2* and *ACR3*, and the role of additional proteins in *ACR2* and *ACR3* regulation.

In a high-throughput yeast two-hybrid screen of predicted coiled-coil motif interactions in the *S. cerevisiae* proteome, the protein Etp1 (encoded by *YHL010c*) was found to interact physically with the AP-1-like transcription factors Yap8, Yap1 and Yap6 (Wang *et al*. 2012). Yap1 controls expression of genes encoding antioxidant and protective functions in response to oxidative and metal stress (Kuge and Jones 1994; Wu and Moye-Rowley 1994; Gasch *et al*. 2000; Wysocki *et al*. 2004; Thorsen *et al*. 2007), whereas Yap6 overexpression confers resistance to cisplatin (Furuchi *et al*. 2001) and to high concentrations of NaCl and LiCl (Mendizabal *et al*. 1998). Etp1 is a 67 kDa large cytoplasmic protein of unknown function. A number of amino acid residues within Etp1 are phosphorylated or ubiquitinated but the functional relevance of these modifications is unknown. Expression of the *ETP1* gene is induced during amino acid starvation conditions and during the transition from fermentative growth to glycerol-based respiratory growth (Cherry *et al*. 2012). It has been shown that Etp1 affects transcription of certain genes (*ENA1*, *HSP12*, *HSP26*) and turnover of specific proteins (Nha1, Hxt3) during ethanol stress and that the *etp1∆* mutant is sensitive to ethanol (Snowdon *et al*. 2009). The human Etp1 homolog BRAP2 is an E3 ubiquitin ligase (Matheny *et al*. 2004) that binds to nuclear localization sequences and BRAP2 may function in retaining proteins in the cytoplasm (Li *et al*. 1998; Asada *et al*. 2004). Etp1 has a zinc finger ubiquitin-binding domain, which is also present in BRAP2, and both proteins can bind ubiquitin (Reyes-Turcu *et al*. 2006). Whether Etp1 functions as an E3 ubiquitin ligase and/or in the regulation of protein localization has not been demonstrated. In this current study, we show that Etp1 protects cells during As(III) stress by affecting *ACR3* expression in a Yap8-independent fashion.

## Materials and methods

### Yeast strains, plasmids and growth conditions

The *S. cerevisiae* strains used in this study are based on BY4742 (MATα *his3Δ1 leu2Δ0 lys2Δ0 ura3Δ0*) (Brachmann *et al*. 1998) and the following mutants from yeast deletion collection (Winzeler *et al*. 1999): *etp1∆* (BY4742 *etp1∆::kanMX*) and *yap8∆* (BY4742 *yap8∆::kanMX*). The *etp1∆ yap8∆* double mutant (BY4742 *etp1∆::kanMX yap8∆::kanMX*) was generated by crossing haploid single mutants using standard procedures. Yeast cells were routinely grown at 30°C on minimal SC (synthetic complete) medium (0.67% yeast nitrogen base; YNB) supplemented with auxotrophic requirements and 2% glucose as a carbon source. Sodium arsenite (NaAsO_2_) (Sigma-Aldrich, St. Louis, MO, USA), sodium arsenate (Na_2_HAsO_4_) (Sigma-Aldrich ) and cycloheximide (Sigma-Aldrich) were added directly to the growth medium. Plate growth assays were performed as previously described (Wysocki *et al*. 2001).

The plasmids used in this study include Yap8-HA under the control of the constitutive *TPI1* promoter in pYX122 (CEN, *HIS3*) (Di and Tamás 2007), GFP-Yap8 controlled by the endogenous *YAP8* promoter in YEp*lac*195 (2μ, *URA3*) (Wysocki *et al*. 2004), p415*TEF1*-10×His-Ub-*LEU2* (pGR295; kindly provided by Gwenaël Rabut), pES15 *ACR3*-*lacZ* (CEN, *URA3*) (Wysocki *et al*. 2004) and pA103 *ACR3* (2µ, *URA3*) (Bobrowicz *et al*. 1997).

### β-Galactosidase activity

Yeast cells expressing the *ACR3*-*lacZ* gene fusion were either untreated or exposed to 0.25 mM As(III) for 6 h. The β-galactosidase activity was measured at least three times in triplicates on permeabilized cells as described previously (Guarente 1983).

### RNA analysis

Cells were grown in synthetic medium during 4 h, then 0.25 mM As(III) was added to the culture and cells collected at 0, 30, 60 and 120 min. RNA extractions were performed with the phenol: chloroform method. Reverse transcription and real-time qPCR (RT-qPCR) were performed as previously described (Sanvisens *et al*. 2014). Real-time PCR was performed under the following conditions: 95°C for 10 s, followed by 40 cycles of 10 s at 95°C and 15 s at 55°C. At the end, a melting-curve analysis was conducted to verify the specificity of the reaction. *IPP1* was used a reference gene for normalization. The primers used were ACR3-F CGCACCGATATACTGACTACGA, ACR3-R ACGGGAAGAAGGCACATAGA, IPP1-F TTACACTGGTCAAGTCAAG and IPP1-R ATCGTTAATATCAATGGCAATA. The comparative threshold cycle (CT) method for relative quantification (ΔΔCT method) was used to analyze the data. The data and error bars represent the relative average and standard deviations of three independent biological samples.

### Chromatin immunoprecipitation

Chromatin immunoprecipitation (ChIP) was performed as previously described (Litwin *et al*. 2018; Maciaszczyk-Dziubinska *et al*. 2020) in *yap8∆* and *etp1∆ yap8∆* cells transformed with HA-tagged *YAP8* behind the *TPI1* promoter (Di and Tamás 2007) or the empty plasmid. Cells were either untreated or exposed to 0.5 mM As(III) for 30 min, and sheared chromatin was immunoprecipitated using anti-HA antibody (H6908, Sigma-Aldrich, 1:2500 dilution) overnight followed by incubation with sepharose protein G beads (Dynabeads Protein G, Life Technologies, Carlsbad, CA, USA). Quantitative PCR (qPCR) was performed using both immunoprecipitates and input samples as templates, a 2× PCR Master Mix SYBR Kit (A&A Biotechnology, Gdansk, Poland) and the CFX Connect Real-Time PCR System (Bio-Rad, Hercules, CA, USA) in a total volume of 15 μl. Primers used for qPCR are PRACR3-for TTACGCTTGCTGGATTGTCA and PRACR3-rev CGTTGCCGCTAAAGTTGATT for the *ACR3* promoter and IPP1-for CTTTATTGGATGAAGGTGA and IPP1-rev TTAATTGTTTCCAGGAGTC for the *IPP1* reference gene. Amplification conditions were as follows: 1 min at 95°C; 40 cycles of 10 s at 95°C; 15 s at 60°C; and 20 s at 72°C. The percentage (% input) value for each sample was calculated according to the formula: ΔCT [normalized ChIP]  =  CT [ChIP] − {CT [Input] − log2 (dilution factor)} and Input %  =  100/2ΔCT [normalized ChIP]. The % input value represents the enrichment of protein at the specific locus and is normalized to the *IPP*1 reference gene. ChIP experiment were performed at least three times. qPCRs were performed two times for each sample and error bars indicate ± standard deviations.

### Yap8 stability

To determine Yap8 protein stability, yeast cells were either untreated or exposed to 0.5 mM As(III). Then, protein translation was stopped by the addition of cycloheximide (CHX) to a final concentration of 50 µg/ml. Cells were harvested at the indicated time points and protein extracts were obtained using the alkali method. Similar amounts of protein were resolved on SDS–polyacrylamide gels and transferred to nitrocellulose membranes. The primary antibodies used include anti-HA (H9658, Sigma-Aldrich, dilution 1:5000) and anti-Pgk1 (22C5D8, Thermo Fisher Scientific, Waltham, MA, USA, dilution 1:10 000) and their appropriate HPR-conjugated secondary antibodies. Immunoblots were scanned using LAS-100 image reader (Fujifilm, Minato, Japan).

### Yeast chromatin fractionation and Yap8 localization

Yeast chromatin fractionation was carried out as previously described (Keogh *et al*. 2006; Oh *et al*. 2018), with slight modifications. One hundred milliliter (OD_600 _= 0.8) yeast cells (*yap8∆* and *etp1∆ yap8∆*) expressing Yap8-HA (pYX122-*YAP8*) or the empty plasmid (pYX122) either untreated or exposed to 0.5 mM As(III) for 30 min were collected, then washed successively with 10 ml distilled water, 10 ml SB (1 M Sorbitol, 20 mM Tris–Cl pH 7.5), 1.5 ml PSB (20 mM Tris–Cl pH 7.5, 2 mM EDTA, 100 mM NaCl, 10 mM β-Mercaptoethanol) and 1.5 ml SB, then resuspended with 1 ml SB. Yeast cell walls were digested by the addition of 125 µl Zymolase (10 mg/ml, Seikagaku, Japan) for 1 h at room temperature. After the digestion, 1 ml ice-cold SB was added, then spheroplasts were collected by gentle centrifugation (2K, 5 min, 4°C) and washed once with 1 ml ice-cold SB. The spheroplasts were resuspended in 500 μl EBX (20 mM Tris–Cl pH 7.5, 100 mM NaCl, 0.25% Triton X-100, 15 mM β-Mercaptoethanol), and then 2.8 μl 100% Triton X-100 was added to lyse the outer cell membrane. Cells were placed on ice for 10 min with occasional mixing. 50 µl of cells were taken and aliquoted as ‘Total’. 1 ml NIB (20 mM Tris–Cl pH 8.0, 100 mM NaCl, 1.2 M Sucrose, 15 mM β-Mercaptoethanon) was layered over the remainder, then centrifuged for 15 min at 12K, 4°C. The upper layer (50 µl) was taken as the ‘Cytoplasmic‘ fraction. Glassy, white nuclear pellets were resuspended in 500 μl EBX, then 5.6 µl 100% Triton X-100 was added to the resuspended nuclear pellets. The resuspended nuclear pellets were incubated on ice for 10 min with gentle mixing every few minutes. Chromatin was pelleted by centrifugation (15 K, 10 min, 4°C). The supernatant was stored as the ‘Chromatin’ fraction. The chromatin fraction was resuspended by 100 µl Laemmli sample buffer then boiled (5 min) for western blot experiments. All fractions were resolved on 12% SDS–PAGE, blotted on nitrocellulose membranes and probed with anti-HA (6908, Merck, Darmstadt, Germany, 1:5000 dilution), anti-H2A (07-146, Merck, Darmstadt, Germany, 1:1000 dilution) or anti-glucose-6-phosphate dehydrogenase (G6PDH; A9521-1VL, Merck, Darmstadt, Germany, 1:10 000 dilution) antibodies. Chemiluminescence signal detection was performed using the Bio-Rad ChemiDoc MP System and Image Lab software (Bio-Rad, Hercules, CA, USA).

### Ubiquitin pulldown assay

Ubiquitinated proteins were purified from yeast cells (*yap8∆* and *etp1∆ yap8∆*) expressing Yap8-HA (pYX122-YAP8) and N-terminally 10× His-tagged ubiquitin (p415*TEF1*-10×His-Ub-*LEU2* (pGR295) derived from pGR140 (Rabut *et al*. 2011; kindly provided by Gwenaël Rabut) using a protocol adapted from (Hovsepian *et al*. 2016). Yeast untreated or exposed to 0.5 mM As(III) for 30 min were grown in SC-leu^–^,his^–^ to OD_600 _= 0.7. One hundred milliliter cells were collected, washed with cold 10 ml distilled water and then resuspended with 500 µl 10% trichloroacetic acid (TCA), incubated for 10 min on ice and then harvested by centrifugation. Pellet was resuspended in 200 µl 10% TCA and lysed using glass beads (Sigma-Aldrich) in a FastPrep FP120 (Thermo Fischer Scientific) for 2 × 30 s at 6K then harvested by centrifugation (13K, 10 min, 4°C). To neutralize the residual TCA present in the pellet 30 μl of 1 M Tris (non-buffered) was added, then pellet was resuspended in 1 ml buffer A (6 M GdnHCl, 20 mM Tris–HCl pH 8, 100 mM NaCl, 20 mM imidazole, 0.1% (v/v) Triton X-100, 100 mM K_2_HPO_4_) and lysates were solubilized at room temperature for 10 min with overhead rotation and harvested by centrifugation (13K, 5 min, RT). Twenty-five microliters of the lysates were taken as the ‘Input’ fraction, diluted with 1.35 ml water, precipitated with 150 µl 100% trichloroacetic acid and resuspended in 25 µl Sample buffer (250 mM Tris–HCl pH 6.8, 500 mM dithiothreitol, 10% SDS, 0.01%, bromophenol blue, 50% glycerol). Remaining samples were incubated with overhead rotation for 2 h at room temperature with Ni-NTA beads (Qiagen, Hilden, Germany) pre-equilibrated 2× with buffer A. The beads were transferred to a Costar (Thermo Fisher Scientific) chromatography column then washed with 2x buffer A, 3x with wash 1 buffer (20 mM Tris–HCl pH 8, 1 M NaCl, 20 mM imidazole, 0.1% (v/v) Triton X-100, 100 mM K_2_HPO_4_) and 3x with wash 2 buffer (20 mM Tris–HCl pH 8, 1 M NaCl, 10 mM imidazole, 0.1% (v/v) Triton X-100, 20 mM K_2_HPO_4_). 10× His-ubiquitin conjugates were finally eluted with 100 μl elution buffer (20 mM Tris–HCl pH 8, 100 mM NaCl, 500 mM imidazole, 100 mM K_2_HPO_4_). Seventeen microliters of eluate was taken as the ‘Eluate’ fraction and resuspended in Sample buffer. All fractions (‘Input’ and ‘Eluate’) were denatured at 95°C for 10 min. Five microliters of the ‘Input’ fraction and 10 μl of the ‘Eluate’ fraction were analyzed by SDS–PAGE (in 4–20% Mini-PROTEAN^®^ TGX Precast Protein Gels, Bio-Rad), transferred on nitrocellulose membrane using Trans-Blot^®^ Turbo Transfer System (Bio-Rad) and immunoblotting with antibodies against the HA-tag (6908, Merck, Darmstadt, Germany, 1:5000 dilution). The level of ubiquitin conjugates was assessed with anti-ubiquitin (P4D1 HRP conjugate, sc-8017, lot #B0817, Santa Cruz Biotechnology, Dallas, TX, USA, 1:1000 dilution) antibodies. Chemiluminescence signal detection was performed using the Bio-Rad ChemiDoc MP System and Image Lab software (Bio-Rad, Hercules, CA, USA).

## Results

### Cells lacking *ETP1* are sensitive to As(III) and As(V)

**Figure 1.**
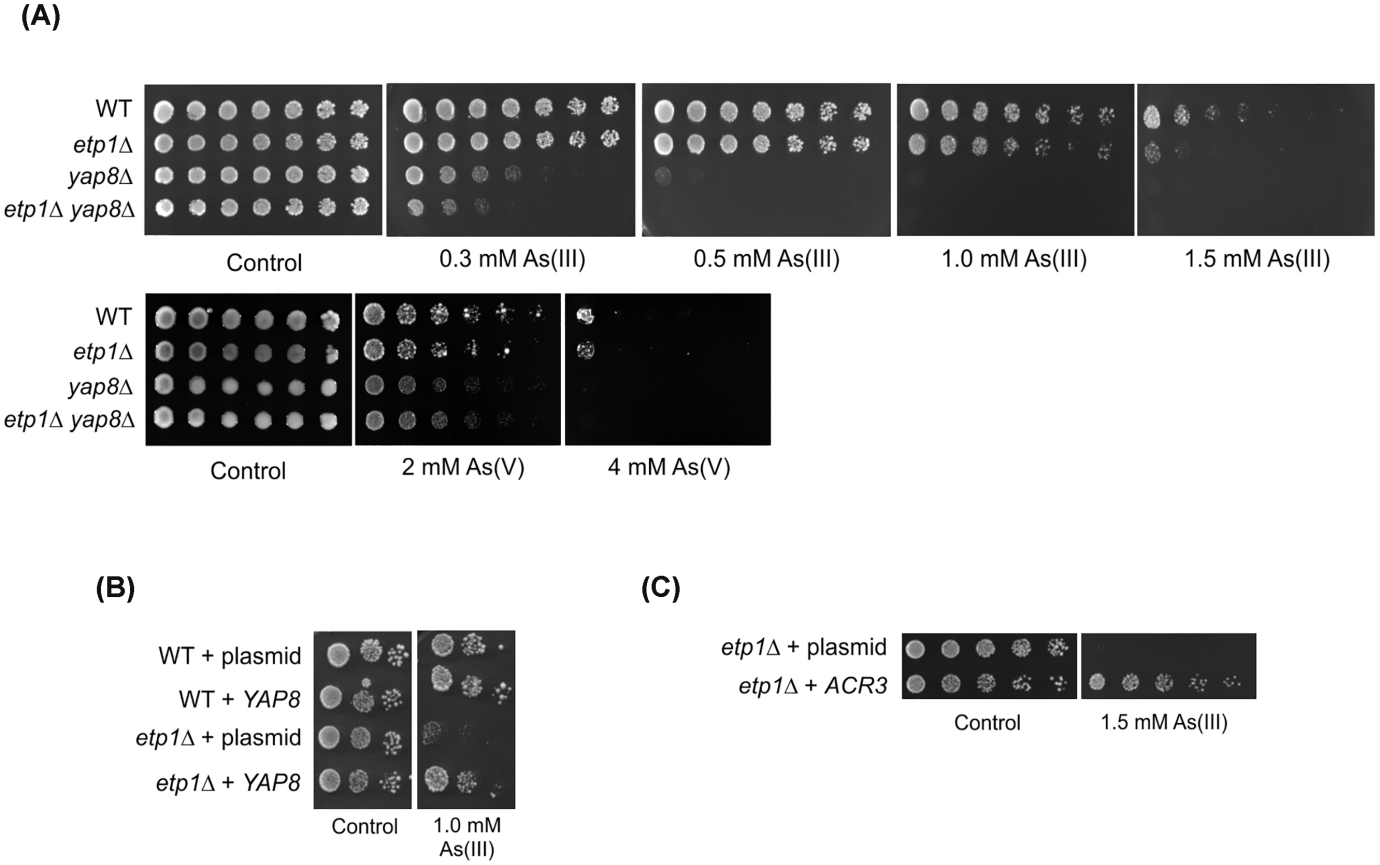
The *etp1Δ* mutant is sensitive to As(III). **(A)** Ten-fold serial dilutions of the indicated strains were plated onto YNB agar plates with or without As(III) and As(V) at the indicated concentrations. Growth was monitored after 2–3 days at 30°C. Growth assays were performed with at least two biological replicates and a representative image is shown. **(B)***YAP8* overexpression improves As(III) resistance in the *etp1Δ* mutant. An empty plasmid or a plasmid carrying GFP-tagged *YAP8* (endogenous *YAP8* promoter, episomal plasmid) was transformed into the indicated strains and growth assays performed as above. **(C)***ACR3* overexpression improves As(III) resistance in the *etp1Δ* mutant. Growth assays were performed as above with the indicated strains transformed with an empty plasmid or a plasmid carrying *ACR3* behind its endogenous promoter on a multicopy plasmid.

Since Etp1 was shown to interact with Yap8 in a yeast two-hybrid screen of predicted coiled-coil motif interactions (Wang *et al*. 2012), we tested whether deletion of *ETP1* is needed for growth in the presence of arsenic, i.e. a condition that requires Yap8 function. As demonstrated previously, *yap8Δ* cells were sensitive to As(III) and As(V) (Menezes *et al*. 2004; Wysocki *et al*. 2004). The *etp1Δ* mutant showed sensitivity to high concentrations of As(III) (Fig. 1A). The *etp1Δ* mutant was also somewhat sensitive to As(V) but the growth defect was modest (Fig. 1A). We conclude that Etp1 is required for optimal growth in the presence of As(III).

### *ACR3* expression is affected in *etp1Δ *cells

**Figure 2.**
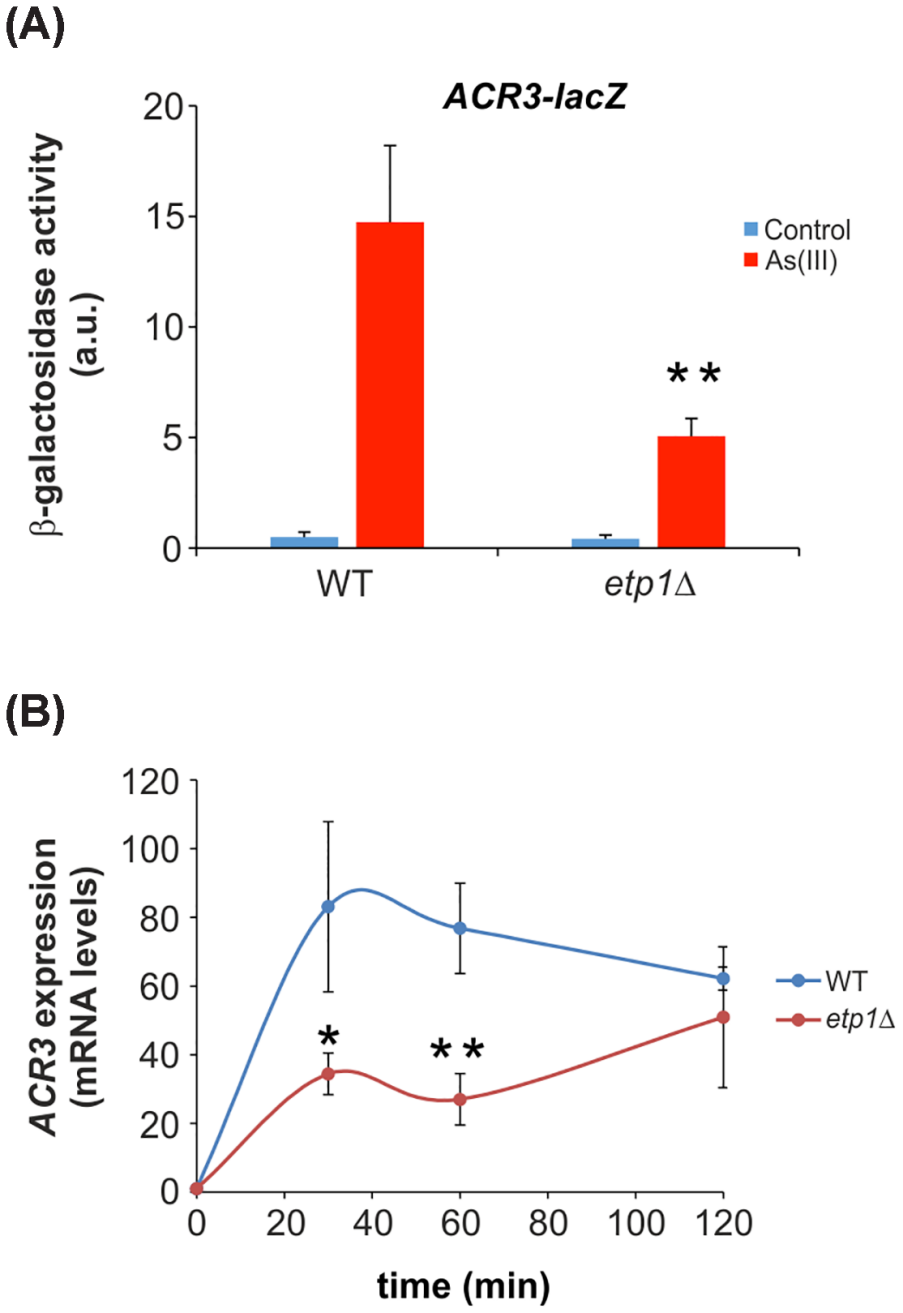
*ACR3* expression is lower in As(III)-exposed *etp1Δ* cells. **(A)** β-Galactosidase activity driven by the *ACR3*-promoter-*lacZ* fusion construct was measured in the indicated strains. Cells were exposed to 0.25 mM As(III) for 6 h or left untreated for the control. The values are the means of three biological replicates performed in triplicate ± standard deviation (SD). *P < 0.05 and **P < 0.005. **(B)** Cells were exposed to 0.25 mM As(III) and samples for RNA extraction were taken at the indicated time points followed by qPCR as described in the 'Materials and Methods' section. *ACR3* expression was normalized to *IPP1* expression. Values are the means of three biological replicates ± SD. *P < 0.05 and **P < 0.005.

As(III) is exported via the plasma membrane transporter Acr3 and a failure to appropriately induce *ACR3* expression leads to arsenic sensitivity (Wysocki, Bobrowicz and Ulaszewski 1997; Wysocki *et al*. 2004). We addressed whether *ACR3* expression is affected in the *etp1Δ* mutant using an *ACR3*-*lacZ* reporter assay. Indeed, As(III)-induced β-galactosidase activity was nearly 3-fold lower in *etp1Δ* cells compared to the wild type (Fig. 2A). To substantiate this, we measured *ACR3* mRNA levels during As(III) exposure using qPCR. Induction of *ACR3* expression occurred with a lower rate in *etp1Δ* cells and reached only ∼60% of the maximal induction level in wild-type cells after 2 h of exposure (Fig. 2B). Thus, *ACR3* expression is clearly compromised in *etp1Δ* cells. A lower amount of Acr3 in the plasma membrane likely explains the observed As(III) sensitivity of *etp1Δ*. To corroborate this, we overexpressed *YAP8* since overexpression of *YAP8* has been shown to result in increased *ACR3* expression (Di and Tamás 2007). As predicted, overexpression of *YAP8* enhanced As(III) resistance of the *etp1Δ* mutant (Fig. 1B). Likewise, overexpression of *ACR3* mitigated the As(III) sensitivity of *etp1Δ* (Fig. 1C). Taken together, these results support the notion that *ACR3* expression is affected in *etp1Δ* cells and that reduced *ACR3* expression accounts for the As(III) sensitivity of *etp1Δ*.

### Yap8 ubiquitination and stability are unaffected by *ETP1* deletion

**Figure 3.**
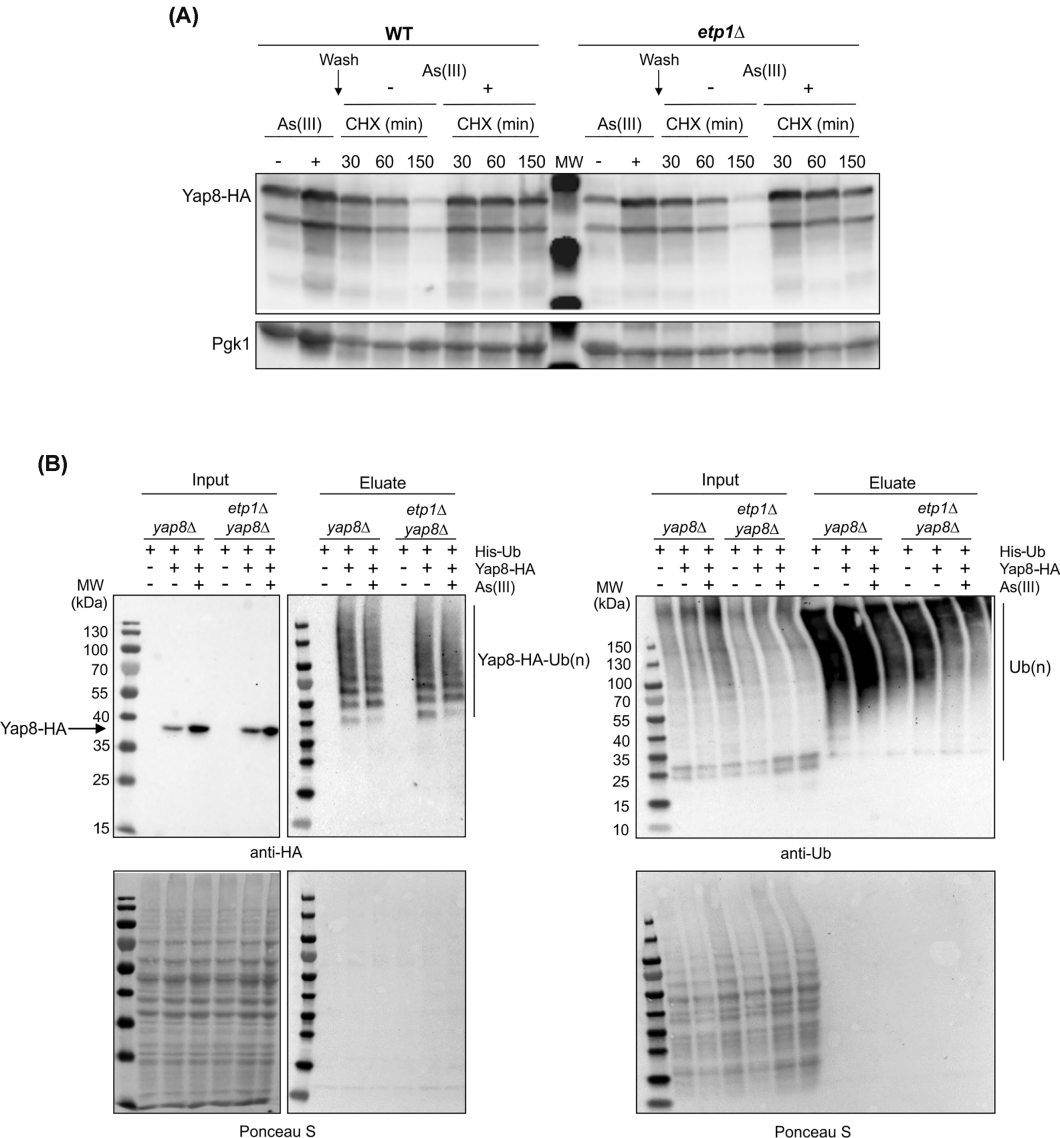
Etp1 does not regulate Yap8 ubiquitination and stability. **(A)** Yap8 stability is unaffected in *etp1Δ* cells. Yap8-HA was expressed from the constitutive *TPI1* promoter in the wild type and the *etp1Δ* mutant and samples were taken for SDS–PAGE at the indicated time points. Cells were exposed to 0.5 mM As(III) for 1 h, then washed and placed in growth medium with or without As(III) as indicated. Cycloheximide (50 µg/ml CHX) was added as indicated to inhibit *de novo* protein synthesis. Immunoblotting was performed with anti-HA and anti-Pgk1 as loading control. The assay was performed with at least two biological replicates and a representative image is shown. **(B)** Yap8 ubiquitination is unaffected in *etp1Δ* cells. Ubiquitination of Yap8-HA was monitored in the indicated cells expressing 10× histidine (His)-tagged ubiquitin. Total cell extracts and ubiquitin conjugates eluted after immobilized-nickel affinity chromatography were separated by SDS–PAGE followed by immunoblotting with anti-HA and anti-ubiquitin antibodies. The ubiquitin pulldown assay was performed with at least two biological replicates and a representative image is shown.

Since Yap8 is the only transcription factor known to regulate *ACR2* and *ACR3* expression (Ilina *et al*. 2008), we asked whether Etp1 affects *ACR3* expression by regulating Yap8. Previous studies showed that Yap8 turnover is regulated at the posttranslational level (Di and Tamás 2007; Ferreira, Menezes and Rodrigues-Pousada 2015). Yap8 levels are low in untreated cells due to degradation via the ubiquitin-proteasome pathway (Di and Tamás 2007) involving the E2 ubiquitin-conjugating enzyme Ubc4 (Di and Tamás 2007) and the E4 ubiquitin ligase Ufd2 (Ferreira, Menezes and Rodrigues-Pousada 2015). Yap8 is stabilized during As(III) exposure resulting in elevated Yap8 protein levels and *ACR3* expression (Di and Tamás 2007). We first analyzed Yap8 stabilization by monitoring the levels of an HA-tagged version of Yap8 in wild-type and *etp1Δ* cells before and after addition of As(III). Yap8-HA is fully functional and complements the As(III) sensitivity of *yap8Δ* and restores the ability to induce *ACR3* expression in the *yap8Δ* mutant (Di and Tamás 2007). Yap8 protein levels increased similarly during As(III) exposure in wild-type and *etp1Δ* cells (Fig. 3A). We next monitored Yap8 half-life after transferring back the cells to As(III)-free medium in the presence of the protein synthesis inhibitor cycloheximide. The half-life of Yap8 appeared unaffected in *etp1Δ* cells compared to that in wild-type cells, both in the absence and presence of As(III) (Fig. 3A). Since Etp1 possesses a zinc finger ubiquitin-binding domain and binds ubiquitin (Reyes-Turcu *et al*. 2006), we reasoned that Etp1 might affect Yap8 ubiquitination. To address this, we immunoprecipitated Yap8-HA from untreated and As(III) exposed cells and analyzed the presence of ubiquitin conjugates by western blot. Yap8 was ubiquitinated both in the absence and presence of As(III), manifested by the occurrence of several slow-migrating bands on the western blot (Fig. 3B). Whether the slow-migrating forms of Yap8 represent polyubiquitination of specific lysine residue(s) or whether Yap8 is monoubiqutinated on several lysine residues is unknown. Notably, Yap8 ubiquitination appeared unaffected in cells lacking *ETP1* (Fig. 3B). Hence, Yap8 ubiquitination and stability are not regulated by Etp1.

### Yap8 nuclear localization and promoter association are unaffected by *ETP1* deletion

**Figure 4.**
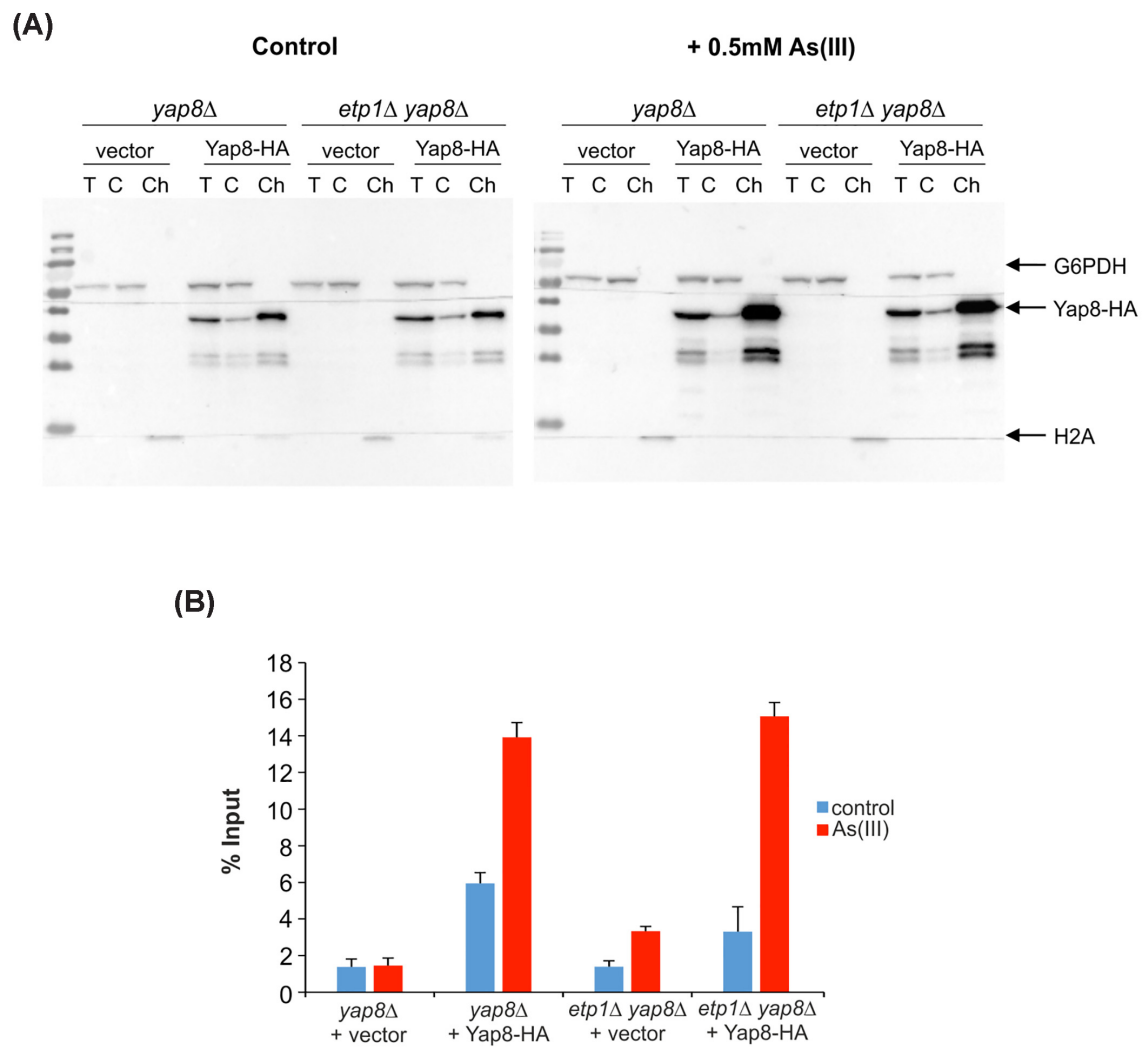
Etp1 does not regulate Yap8 nuclear localization and *ACR3* promoter association. **(A)** Nuclear localization of Yap8 is unaffected in *etp1Δ* cells. Yeast chromatin was fractionated as described in the 'Materials and Methods' section and the presence of Yap8-HA was monitored by immunoblotting. T, total extract; C, cytosolic fraction; Ch, chromatin fraction. Immunoblotting was performed with antibodies against the HA-tag, histone H2A as a marker for the chromatin fraction and glucose-6-phosphate dehydrogenase (G6PDH) as a marker for the cytosolic fraction. The assay was performed with at least two biological replicates and a representative image is shown. **(B)***In vivo* occupancy of Yap8 on the *ACR3* promoter is unaffected in *etp1Δ* cells as determined by ChIP. The indicated cells carrying Yap8-HA or the empty vector were either untreated (control) or exposed to 0.5 mM As(III) for 30 min, and qPCR was performed on chromatin fragments isolated after immunoprecipitation using an anti-HA antibody as described in the 'Materials and Methods' section. Data from three biological replicates are shown, and the error bars represent standard deviations.

We previously demonstrated that Yap8 resides in the nucleus where it constitutively binds to the *ACR2*-*ACR3* promoter as a homodimer, and that the presence of As(III) does not impact Yap8 homodimerization, nuclear localization or promoter association (Wysocki *et al*. 2004; Di and Tamás 2007; Kumar *et al*. 2016). In contrast, another group reported that As(III) exposure results in the translocation of Yap8 from the cytoplasm to the nucleus (Menezes *et al*. 2004). The reason for this discrepancy is not clear. Nevertheless, the fact that the human Etp1 homolog BRAP2 binds to nuclear localization sequences (Li *et al*. 1998; Asada *et al*. 2004) raised the possibility that Etp1 might affect Yap8 localization. To address this, we analyzed Yap8-HA localization by biochemically isolating yeast nuclei followed by western blotting. In agreement with our earlier results (Wysocki *et al*. 2004; Kumar *et al*. 2016), Yap8-HA localization was predominantly nuclear and unaffected by As(III) (Fig. 4A). Importantly, the absence of Etp1 did not affect Yap8 nuclear localization, neither in the absence or presence of As(III) (Fig. 4A). We next addressed the *in vivo* association of Yap8 with the *ACR3* promoter by ChIP assays. The ChIP indicated no major impact of *ETP1* deletion on Yap8-HA occupancy on the *ACR3* promoter (Fig. 4B), neither in the absence or presence of As(III). We conclude that nuclear localization and promoter association of Yap8 *in vivo* are unaffected by *ETP1* deletion.

### Etp1 confers As(III) resistance independently of Yap8

The results above indicated that Etp1 does not regulate Yap8. To test whether Etp1 confers As(III) resistance independently of Yap8, we compared growth of the *etp1Δ yap8Δ* double mutant to that of the single mutants. The *yap8Δ* single mutant was highly As(III) sensitive as expected, and the *etp1Δ yap8Δ* double mutant was somewhat more As(III) sensitive than the *yap8Δ* single mutant (Fig. 1A). The additive As(III) sensitivity of *ETP1* deletion in *yap8Δ* cells was similar to that of *ETP1* deletion in wild-type cells, suggesting that Etp1 confers As(III) resistance independently of Yap8. Additional deletion of *ETP1* did not further sensitize *yap8Δ* cells to As(V) (Fig. 1A), supporting the notion that Etp1 function is important primarily during As(III) stress.

## Discussion

Previous work implicated Etp1 in transcriptional activation of *ENA1*, encoding an Na^+^ and Li^+^ efflux protein, during salt and ethanol stress as well as ethanol stress-dependent induction of the heat shock genes *HSP12* and *HSP26* (Snowdon *et al*. 2009). Etp1 also affected the turnover of the Na^+^/H^+^ symporter Nha1 and the hexose transporter Hxt3 during ethanol stress as more Nha1 and Hxt3 were present in cells lacking *ETP1*. Additionally, Etp1 is required for optimal growth on ethanol-containing medium whereas it is dispensable for resistance to salt stress (LiCl and NaCl) (Snowdon *et al*. 2009). Here, we showed that Etp1 contributes to As(III)-induced *ACR3* expression and confers As(III) resistance. Reduced *ACR3* expression is likely to account for the As(III) sensitivity of *etp1Δ* since As(III) export via Acr3 is critical for resistance (Bobrowicz *et al*. 1997; Wysocki, Bobrowicz and Ulaszewski 1997; Wysocki *et al*. 2004). Thus, Etp1 appears to impact gene expression under various stress conditions. How does Etp1 affect transcription? Etp1 has a cytosolic localization and no obvious DNA-binding domain (Snowdon *et al*. 2009). Hence, Etp1 is not likely to directly bind DNA and act as a transcription factor. Instead, Etp1 might affect transcription indirectly. The human Etp1 homolog BRAP2 binds to nuclear localization sequences and regulates the localization of some nuclear proteins (Li *et al*. 1998; Asada *et al*. 2004; Fulcher *et al*. 2010). Hence, Etp1 might affect the localization of transcriptional regulators in yeast in analogy to BRAP2. Moreover, BRAP2 is an E3 ubiquitin ligase (Matheny *et al*. 2004) and like BRAP2, Etp1 possess a zinc finger ubiquitin-binding domain and can bind ubiquitin (Reyes-Turcu *et al*. 2006). The presence of polyubiquitin chains dictates the fate of proteins, such as the degradation of proteins with K48-linked ubiquitin chains attached, by the proteasome (Swatek and Komander 2016; Yau and Rape 2016). Whilst Etp1 affected turnover of specific proteins (Snowdon *et al*. 2009), it remains to be demonstrated whether Etp1 functions as an E3 ligase and whether transcriptional regulators are among its substrates. In this current study, we showed that Yap8 ubiquitination, stability, nuclear localization and *ACR3* promoter association were unaffected in *etp1Δ* cells. Thus, Etp1 affects *ACR3* expression independently of Yap8. This was corroborated by genetic data indicating that Etp1 confers As(III) resistance independently of Yap8. Moreover, we found no interaction in a dedicated yeast two-hybrid assay between full-length Yap8 (bait) and full-length Etp1 (prey). Thus, it appears that the interaction between the predicted coiled-coil regions in Etp1 (residues 487–552) and Yap8 (residues 20–75) (Wang *et al*. 2012) does not occur with the full-length proteins. How can Etp1 affect *ACR3* expression independently of Yap8 given that Yap8 is the only transcription factor known to control *ACR2* and *ACR3* expression (Wysocki *et al*. 2004; Ilina *et al*. 2008)? Recent studies have shown that coactivator complexes and chromatin remodeling factors are implicated in proper induction of *ACR3* expression during As(III) stress (Menezes *et al*. 2017; West *et al*. 2019). It is tempting to speculate that Etp1 affects *ACR3* mRNA levels by regulating localization and/or turnover of component(s) of these coactivator complexes and chromatin remodeling factors.

To sum up, our data indicate that Etp1 affects *ACR3* expression and confers As(III) resistance in a Yap8-independent fashion. Thus, Etp1 is a novel arsenic resistance factor that impacts gene expression under various stress conditions; the mechanistic details remain to be elucidated. Since Etp1 is a putative E3 ligase, future efforts should be directed toward demonstrating its ubiquitin ligase activity and to identify its physiological substrates.

## Acknowledgments

We dedicate this paper to our dear colleagues Dr Stefan Hohmann (1956–2021) and Dr Christer Larsson (1958–2021) and thank them for their contributions to science and the yeast community in Gothenburg and internationally, and for great collaborations and friendship over the years. We thank Peter Dahl (University of Gothenburg) for expert technical assistance constructing the *etp1Δ yap8Δ *strain, Ewa Błaszczak (University of Wroclaw) for technical assistance with the ubiquitin pulldown assay and Gwenaël Rabut (Université de Rennes) for providing the pGR295 plasmid.
